# Optimization of the Acquisition Time and Injected Dose of ^18^ F-Fluorodeoxyglucose Based on Patient Specifications for High-Sensitive Positron Emission Tomography/Computed Tomography Scanner

**DOI:** 10.1055/s-0043-1771284

**Published:** 2023-09-06

**Authors:** Murtadha Al-Fatlawi, Farideh Pak, Saeed Farzanefar, Yalda Salehi, Abbas Monsef, Peyman Sheikhzadeh

**Affiliations:** 1 Radiological Techniques Department, AL-Mustaqbal University College, Babel, Iraq; 2 Department of Radiation Oncology, Washington University in St. Louis, St. Louis, United States; 3 Department of Nuclear Medicine, Imam Khomeini Hospital Complex, Tehran University of Medical Sciences, Tehran, Iran; 4 Department of Radiology, Center for Magnetic Resonance Research, University of Minnesota Medical School, Minneapolis, United States; 5 Department of Radiation Oncology, University of Minnesota Medical School, Minneapolis, United States

**Keywords:** dose optimization, BGO-based positron-emission tomography, dependency, reconstruction algorithm

## Abstract

**Background** This study was aimed to optimize the fluorodeoxyglucose (FDG)-administered dose and scan time based on patient specifications using a highly sensitive five-ring bismuth germanium oxide (BGO)-based positron emission tomography/computed tomography (PET/CT) scanner (Discovery IQ).

**Methods** We retrospectively analyzed 101 whole-body ^18^ F-FDG PET/CT images. Patient data were reconstructed using ordered subset expectation maximization with resolution recovery algorithms (OSEM + SharpIR). Signal-to-noise ratio (SNR) was calculated for each patient, standardized to SNR _norm_ , and plotted against three body index parameters (weight, body mass index, and lean body mass). Two professional physicians blindly examined image quality at different patient time per bed positions to determine the minimum acceptable quality. To select images of acceptable quality, the noise index parameter was also measured. A new dose-time product (DTP) was established for each patient, and a predicted injected dose was assumed.

**Results** We found an almost linear association between patient weight and normalized SNR, and patient weight had the highest R ^2^ in the fitting. The redesigned DTP can reduce results by approximately 74 and 38% compared with ordinary DTP for 80- and 160-s scan durations. The new dose regimen formula was found to be DTP = *c/t × m* ^1.24^ , where *m* is the patient weight, *t* is the scan time per bed position, and *c* is 1.8 and 4.3 for acceptable and higher confidence states, respectively, in Discovery IQ PET/CT.

**Conclusion** Patient weight is the best clinical parameter for the implementation of ^18^ F-FDG PET/CT image quality assessment. A new dose-time regimen based on body weight was proposed for use in highly sensitive five-ring BGO PET-CT scanners to significantly reduce the injection dose and scan times while maintaining sufficient image quality for diagnosis.

## Background

Positron emission tomography/computed tomography (PET/CT) corroborates anatomical details by providing functional information. With the increasing number of clinical applications of this imaging modality in oncology, patients require PET/CT scans more frequently at various stages, such as initial staging, interim response, response to therapy, and follow-ups. 1 2 3 4 Oncological imaging commonly utilizes an effective radiopharmaceutical, namely ^18^ F-fluorodeoxyglucose ( ^18^ F-FDG). 5 ^18^ F-FDG is a fluorine radioisotope produced by cyclotrons that can be scanned 50 to 75 minutes after injection into a patient's body. 6 The management of the injected dose of ^18^ F-FDG for whole-body PET-CT scans follows the European Association of Nuclear Medicine (EANM) guidelines. 6 The ENAM guidelines ensure that the measured FDG tumor uptake is within specific limits (370–740 MBq), regardless of the type of device used or study location. 6 7 The new version of ENAM guidelines (2015) provides an overview of the earlier findings and attempts to address some new developments in PET scans, such as time-of-flight technology. 6 Few studies have aimed to optimize FDG examinations after the last update of the EANM recommendations. According to a study, PET/CT with a bismuth germanium oxide (BGO) detector can reduce the ^18^ F-FDG injection dose by up to 25% in patients with Hodgkin's lymphoma without sacrificing image quality. 8 Nevertheless, this study focused on a single indication to optimize the administered dose. Another study that used a four-ring lutetium-yttrium-orthosilicate (LYSO) TrueV scanner (Siemens Medical Solutions, Knoxville, TN, United States) assumed that a modest reduction of either injected FDG dose or the time per bed position to levels below the limits provided in the EANM procedure guideline might be possible. 9 In the Fred Wickham study, another Siemens scanner (Biograph mCT Flow) was used to establish an expression in terms of sex, height, and weight to optimize the injected dose and acquisition times. 10 Reconstruction algorithms have been developed over the years to reduce errors and artifacts and improve image quality. Ordered subset expectation maximization (OSEM) is the most widely used algorithm for PET/CT scanning. Advances in OSEM and resolution recovery methods, such as point spread function modeling, have improved PET image quality by considering all statistical and physical processes during data acquisition. 11 12 PET-CT scanners have comparable image quality results depending on the technology used for detecting tumors, in addition to data acquisition and reconstruction methods. The acquisition time and injection dose are influenced by scanner sensitivity. System sensitivity is one of the critical parameters of each scanner, depending on the detector technology, crystal material, and axial field-of-view (FOV) in conventional cylindrical scanners. 13 14 In addition, the detectability of ^18^ F-FDG features in PET/CT scans is influenced by reconstruction algorithms. Therefore, the ^18^ F-FDG guidelines need to be updated to consider different scanner types with different sensitivity and reconstruction algorithms. GE Healthcare has recently manufactured GE Discovery IQ, a highly sensitive long-axial FOV PET scanner based on five-ring BGO detectors with a sensitivity of 22 kcps/MBq. 15 16 In this study, we aimed to optimize the FDG-administered dose based on patient specifications in whole-body scans using this scanner.

## Methods

### Patient

^18^ F-FDG PET/CT scans of 101 patients of both sexes (65 females and 36 males) were randomly selected. The patients were scanned according to standard clinical protocols and guidelines of the EANM in ^18^ F-FDG PET/CT imaging. 6 Body weight of 45 to 113 kg and different clinical indications were included. All studies were performed retrospectively using anonymized clinical patient data. All patients received a dose of approximately 0.1 mci (3.7 MBq) per kilogram of body weight according to the current guidelines of the EANM, as shown in Table 1 . For adherence to the guidelines for patient preparation, the scans were acquired 60 ± 5 minutes after the injection with the patients in the supine position and their arms up. Furthermore, different time per bed positions ranging from 1.3 to 6 minutes in terms of minutes per bed position (mpb) were used for the patients. Based on the GE-recommended protocol, to achieve higher image quality, higher time per bed position was used for weights greater than 60 kg.

**Table 1:** Patient characteristics and acquisition parameters

Number of patients	Weight (kg)		Height (cm)		Prescribed dose (MBq)		Time per bed position (min)	
	Range	Mean ± SD	Range	Mean ± SD	Range	Mean ± SD	Range	Mean ± SD
101 (65 female + 36 male)	45–113	72.2 ± 13	147–183	165 ± 9	114–470	305.7 ± 59	1.3–6	4.6 ± 0.7

### Positron Emission Tomography/Computed Tomography Imaging

All images were scanned using the GE Discovery IQ PET/CT system (General Electric Healthcare, WI, United States), which combines a high-sensitivity PET scanner (22 cps/kBq) and a 16-slice CT scanner (120 kV, 80 mA). 16 A reconstruction algorithm featuring 4 iterations, 12 subsets, and a 6.4-mm Gaussian postprocessing filter with resolution recovery capability (OSEM + SharpIR) was used as a routine reconstruction technique for this system. The 192 × 192 matrix size, resulting in a 3.64 × 3.64 × 3.26-mm pixel size, formed PET images.

### Quantitative Image Analysis

Parameters, such as injected activity, time per bed position, and body weight (kg), were derived for each patient, body mass index (BMI) was calculated, and lean body mass (LBM) was theoretically defined and calculated based on the method presented by Hume. 17 The dose time product (DTP) was obtained using the formula FO, where *A* is the injected activity and *t* is the scan time (time per bed position). The SNR in the liver was selected as an index of image quality because of the relatively homogeneous uptake of FDG. Patients with inhomogeneous uptake mainly due to metastasis or other irregularities in the liver were excluded from this study. A spherical voxel of interest (VOI) 40 mm in diameter was placed in the center of the largest liver axial slice to avoid partial volume effects at the liver edges and separately from the porta hepatis and major vessel area of the liver to target only liver tissues using Amid software (version 1.0.3). The SNR was calculated according to Equation 1:



Mean is the mean pixel value within the VOI.

SD is the standard deviation in the observed region.

**Fig. 1:**
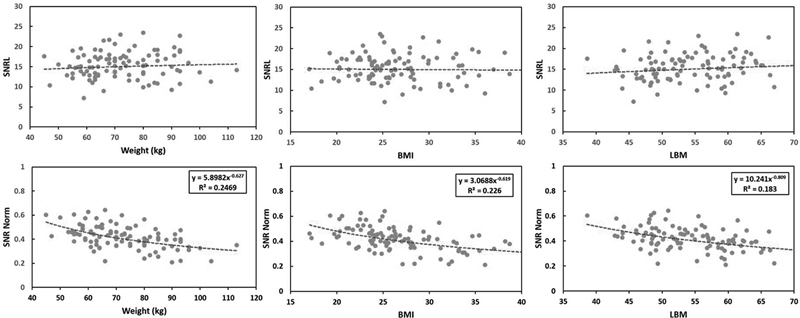
*Top row* : Signal-to-noise ratio of the liver (SNRL) as a function of patient parameters (weight, BMI, and LBM). *Bottom row* : Normalized SNR (SNR norm) for injected dose activity and scan time as a function of patient weight, BMI, and LBM. *Scattered dots* are the SNR norm data fitted to nonlinear regression ( *dotted curve* ).



The result of the equation is reported as SNR liver (SNRL) for each patient. SNRL was normalized to eliminate its dependency on time per bed position for each patient (SNRnorm [MBq·min] ^−1/2^ ) according to Equation 2 and then plotted against different patient parameters, such as weight (kg), BMI, and lean body mass, as shown in Fig. 1 .

Nonlinear fitting was performed on the graph of SNR _norm_ and patient parameters to find *a* and *p* function values in Equation 3, where SNR _fit_ ([ *MBq* min] ^−1/2^ ) is the result of the fit:



*p* is the patient-dependent parameter.

*a* , *d* is the fitting-derived constants.

A combination of Equations 2 and 3 showed that SNRL, and hence, the image quality, was constant if  this constant is equal to the acceptable SNRL (SNR _acc_ ).

### Qualitative Image Evaluation

Two expert nuclear medicine physicians determined the SNR _acc_ for all patient images using each algorithm. SNR _acc_ represents the constant of SNR _L_ corresponding to the highest value of the patient parameters for which the image quality was still acceptable. To achieve this goal, raw image data representing a 96-kg male, 287-MBq injected dose, and 160-s scan time per bed position were selected and then reconstructed using the following different time per bed positions (80, 40, 20,10, and 5 seconds) so that new images with different qualities was generated. All resulting images in the coronal and axial views were evaluated by two expert nuclear medicine physicians (more than 8 years of experience) to select the least acceptable image qualitatively. Consequently, the image that exhibited the lowest acceptable SNR (SNRacc) required for accurate diagnosis was chosen. It should be noted that the physician was blinded to all patient information, such as time per bed position (the reconstructed time) and injection activity. On the contrary, quantitatively, the noise index of all the generated images was measured by obtaining the coefficient of variation (COV% =FO). All images with higher SNR (higher than SNRacc) presented to have a good coefficient of variation, by the same token they were scored a quality level of good or moderate in the clinical visual assessment. A new DTP was calculated according to patient-dependent parameters using Equation 4:



Finally, a new injection activity and time per bed position were calculated using the new DTP value. It's worth to be mentioned that this method was used previously by Groote et al to have similar outcomes, and yet his study was valid only on the Biograph TruePoint PET-CT scanner.

## Results

The measurements and calculations of patient characteristics and parameters are displayed in Table 1 . The graphs in Fig. 1 show the measured SNRL as a function of body weight, BMI, and LBM (according to the theoretical calculations). A linear function was fitted to the scatter data to determine the behavior of SNRL against each patient's parameters.

Fig. 1 demonstrates an almost linear fitting for SNR (SNR _fit_ ), which was achieved by fitting the SNR _norm_ (SNR _L_ after normalization) with all the parameters, which resulted in the determination of the fit parameters *a* and *d* for each patient parameter. The regressions in Fig. 1 were obtained using the data of all samples scanned using our scanner in this study. The R ^2^ values were 0.24, 0.22, and 0.18 for patient weight, BMI, and LBM respectively. Patient weight had the highest R ^2^ and was the easiest parameter to implement in the clinic; therefore, it was chosen as the best parameter for image quality assessment and dose optimization.

**Fig. 2:**
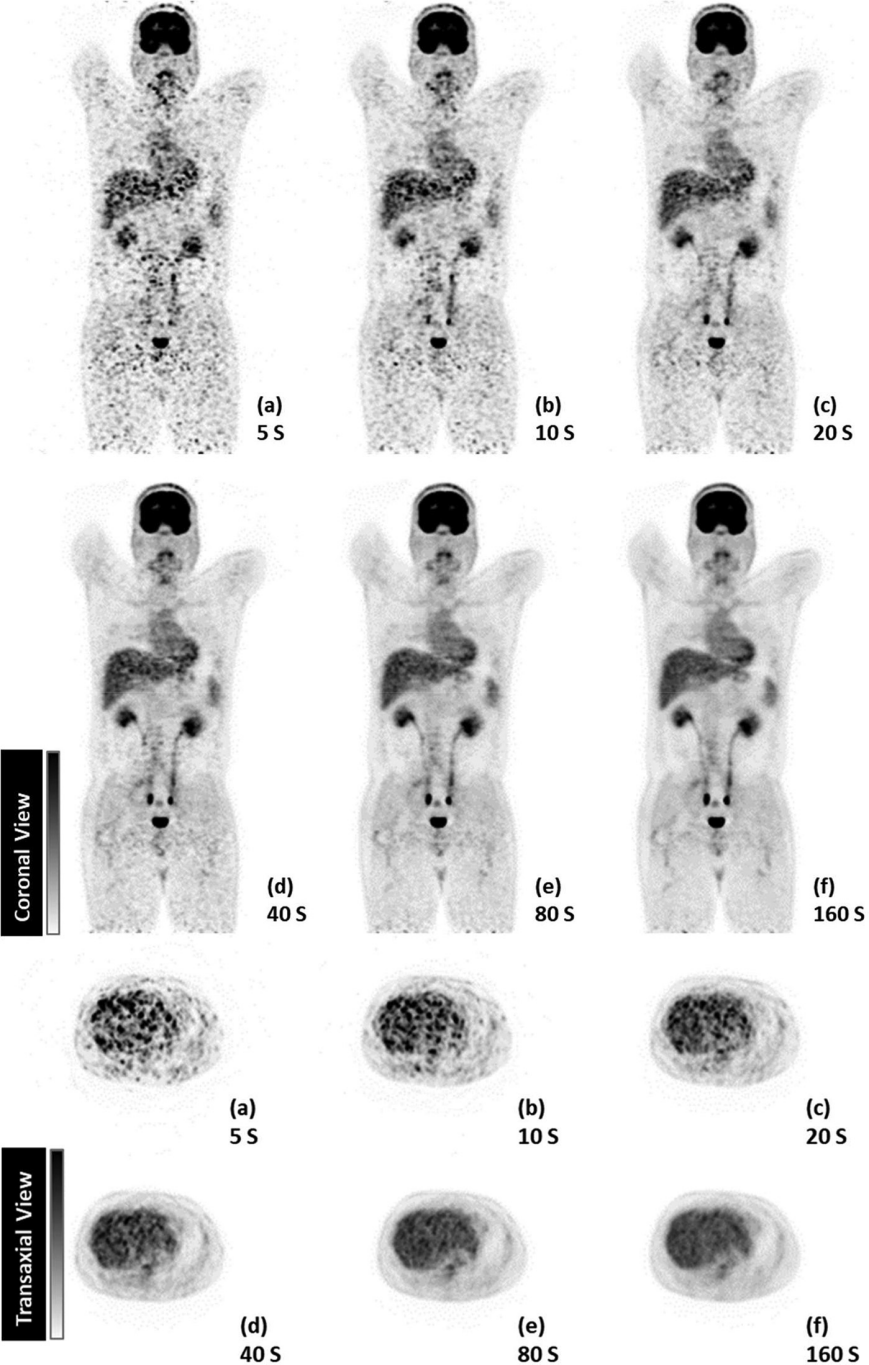
Coronal and transaxial views of a whole-body FDG-PET scan for a 95-kg patient (injected dose = 287 MBq) with different scan times, including 5, 10, 20, 40, 80, 160 s. Scans were performed on the Discovery IQ five-ring PET-CT.

**Fig. 3:**
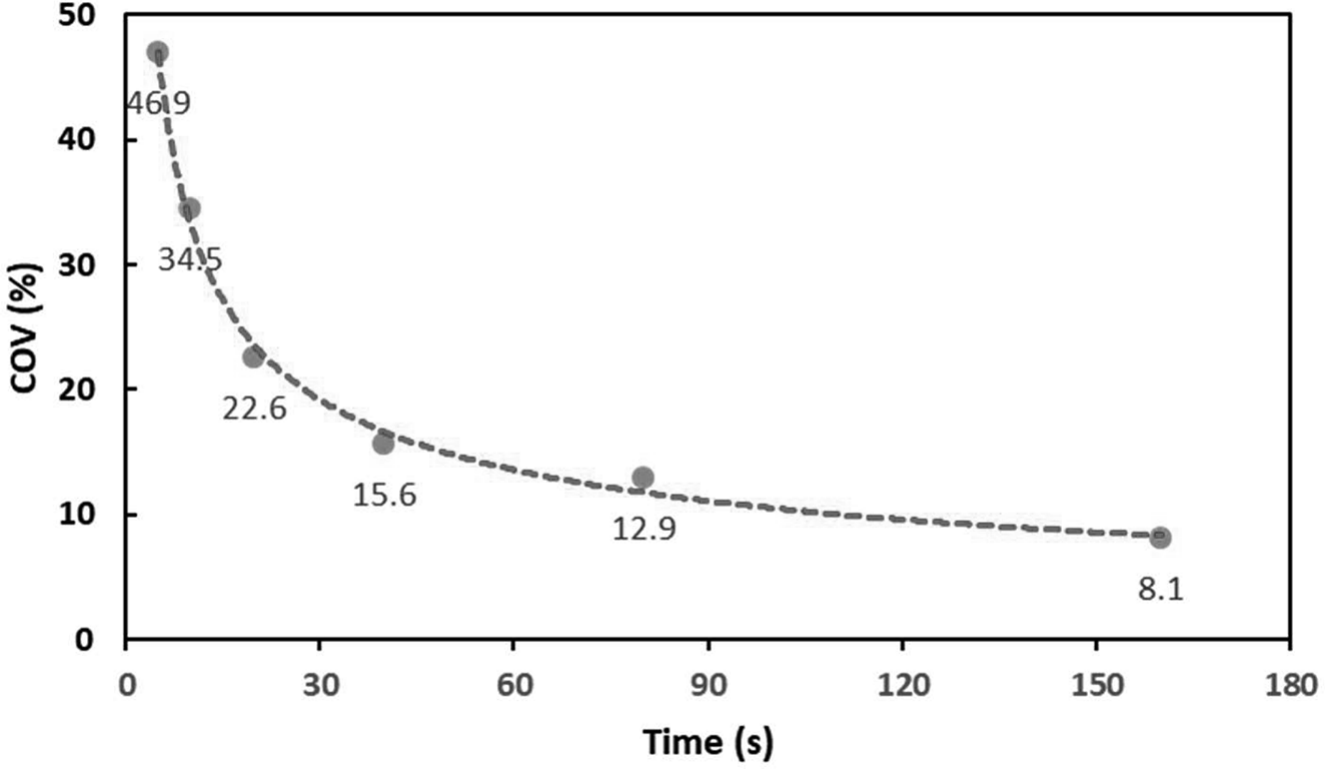
Coefficient of variation (COV) values for the liver of a 96-kg patient in whole-body FDG-PET scan using different scan times. The threshold COV, which provides acceptable image is 15%; therefore, COVs of 12.9% and 8.1% are included as reliable images.

Fig. 2 shows the FDGPET images generated at different time per bed positions in coronal and axial views. All images were shown to the physician and the 80-second duration was selected as the time per bed position required for minimum acceptable quality image; however, 160-second time per bed position provided much higher confidence for the physician when reporting. For these time per bed position images, the SNR was calculated as 7.9 and 12.3 for 80 and 160 seconds, respectively. In addition to qualitative and quantitative assessments, the COV of each image was calculated and plotted. The COVs for 60 and 180 seconds were 12.9 and 8.1%, respectively, and for the other scan time per bed position,COVs were more than 15% ( Fig. 3 ). Appropriate and acceptable COV should be under 15%; 18 therefore, just these two time per bed position 180 and 60 s were included, and the others scan times were excluded.

Table 2 illustrates the process for obtaining a new DTP. The values for the fit parameters *a* and *d* have been shown in Table 2 . A paired-sample *t* -test was used to show a significant difference ( *p* < 0.0001) between the old and new DTP values. Based on the acceptable and high-confidence SNR, the old DTP value was reduced by approximately 74 and 38%, respectively.

**Table 2:** The difference between the new dose time product (DTP) formula in both acceptable- and high-confidence signal-to-noise ratio values and the old DTP formula

Index/parameter	a	d	Fitting equation	SNR acceptable	Dose time Product DTP formula	Diff in DTP%	T test DTP reduction
Weight (acceptable confidence)	5.89	0.62	5.89 X ^−0.62^	7.9	1.8 (weight) ^1.24^	−74.4	( *p* < 0.0001)
Weight (higher confidence)	5.89	0.62	5.89 X ^−0.62^	12.3	4.3 (weight) ^1.24^	−38.9	( *p* < 0.0001)

**Fig. 4:**
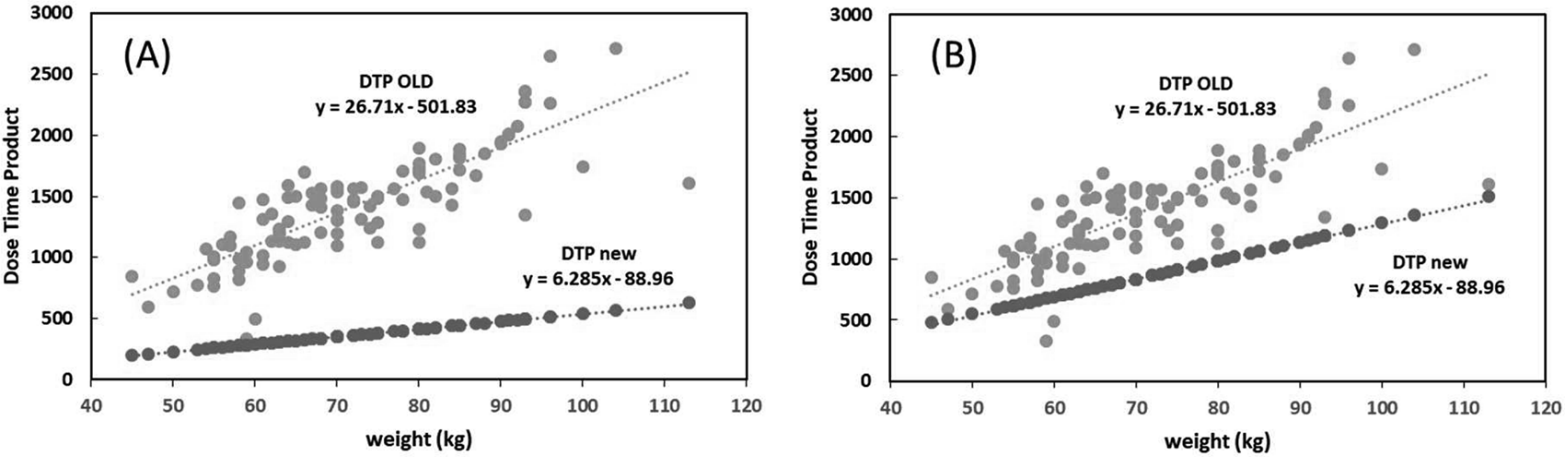
( **A** ) The dose time product (DTP) according to the patient weight following the EANM guidelines and routine clinical procedures ( *gray dots* ) and the new-DTP proposed formula ( *black dots* ) with acceptable confidence. ( **B** ) The DTP according to the patient weight following the EANM guidelines and routine clinical procedures ( *gray dots* ) and the new-DTP proposed formula ( *black dots* ) with higher confidence.

In Fig. 4 , the Wilcoxon matched-pairs signed-rank test showed a significant difference ( *p* < 0.0001) between the old DTP values and the optimized new DTP values. There is a significant reduction ( *p* < 0.0001) in the new optimized DTP compared with the old DTP based on patient weight ( Fig. 4A ). This reduction was more significant for an acceptable SNR. Final new DTP formula (activity × time), depending on the patient weight was obtained as follows:

 MBq for acceptable confidence.

 MBq for higher confidence.

## Discussion

The guidelines for tumor imaging using ^18^ F-FDG show an average injected activity of 370 to 740 MBq. 6 7 However, this dose recommendation does not consider the image reconstruction algorithms used and also states that the dose can be lowered in highly sensitive PET/CT systems. A highly sensitive PET/CT scan, such as a GE Discovery 5-ring BGO-based detector, has shown a significant positive impact on the image quality. Some studies have dealt with dose-time optimization based on patients' physical specifications. Niederkohr et al suggested that using specific equipment, a slight reduction might be possible in the administered FDG dose or the PET scan time per bed positions to levels below the values identified in the EANM/SNMMI procedure guidelines. 9 Wickham et al reported a reduction in the mean activity administered to a group of patients compared with the current protocol with the same consistent image quality. 10 Prieto et al indicated that with ^18^ F-FDG, an injection dose reduction of 23.4% (down to 3.57 MBq/kg) can provide an acceptable image quality. 19 Nevertheless, previous studies have been performed using a four-ring lutetium oxyorthosilicate PET/CT scanner. Dziuk et al reported that ^18^ F-FDG-injected dose could be reduced by up to 25% when using a five-ring BGO crystal PET/CT camera, without substantial impact on image quality. However, this study only considered patients with Hodgkin lymphoma. 8 According to the existing guidelines, advanced PET/CT technology allows for a significant reduction in radiotracer doses. However, these studies were limited by the systems they used and the approach they used was not adopted by other scanners. In this study, images were acquired using a five-ring BGO-based GE discovery-IQ PET/CT scanner. This scanner has a sensitivity of 22 cps/MBq, which is almost three times more sensitive than that of conventional scanners. The high sensitivity of the scanner was achieved using numerous technological modifications, including the three-dimensional mode, an extended axial FOV, and an increase in the number of detector rings from two to five along the FOV. The data in Fig. 1 ( *top row* ) were obtained using a linear relationship between patient parameters and FDG dose in both algorithms, but the scan time per bed position varied for different bodyweight classes. However, Equation 2 can be used to adjust the scan-time adaptation. Based on other findings, 20 SNRL graphs, and the routine EANM guidelines, it was observed that SNR _L_ decreases with increasing body weight and other parameters for other scanners. However, in our scanner, we observed a slight increase for patients weighing more than 60 kg because we increased the scan times based on GE recommendations to prevent image degradation.

For SNRL and SNR norm calculations, the method was based on a study by Groot et al in 2014. The image quality analysis was based on the liver SNR. Furthermore, physiology can also affect the SNR of the patients. Variations in plasma clearance, overweight, and/or plasma glucose levels might affect the biodistribution of FDG and, thus, the SNR. However, this study suggests that these effects are either unusual or not as noticeable as the attenuation. The use of the liver as a reference for image quality in clinical observations and image analysis is an acceptable method. 21 22 23 The liver was chosen because it is the only organ in the body that shows relatively uniform absorption of FDG. SNRL, on the contrary, represents physiological uptake variability. As the only exclusion criterion for this study was the heterogeneity of liver uptake, our findings can be generalized to all FDG whole-body scans if our scanner is used. Normalizing the SNR (the correction process of different times of bed per position and injected activity) is a valid method for quantifying image quality independent of time (minutes) per bed position (mbp), according to Cox et al. 24 Table 2 shows the process of obtaining new DTP values under these two conditions. By applying the value of SNR _acc_ to Equation 4, we ensured that the output (DTPnew) was within the acceptable image quality. The new DTP corresponds to the DTP in the conventional method (FO) but considers patient parameters and is more sensitive to the type of algorithm used for processing images. Cox et al and de Groot et al proposed methods to obtain new DTP values within acceptable image quality for adult patients, depending on their SNR. 20 24 Fitting SNRnorm to different patient-dependent parameters ( *p* ) showed that SNRnom had the strongest relationship with body weight (the highest R ^2^ ). Accordingly, this would lead to a greater influence on the optimized DTP values among the other parameters. Because weight is the simplest patient-dependent parameter and a very practical parameter to use, the choice for body weight was considered to be used in the optimization of the FDG-injected dose. The DTP values were tested with a paired-samples *t* -test, demonstrating a dramatic decrease ( *p* < 0.0001) in the new DTP values in the two states compared with the conventional DTP (DTP before optimization) values. Our proposed formula for injected dose can significantly reduce the dose received by the patient. On the contrary, based on our new DTP, if we want to use conventional injection parameters, we can reduce the scan time, which in turn can decrease the artifacts due to patient movements and increase the PET-CT center throughput.

## Conclusion

Compared to BMI and LBM, patient weight is the best parameter with the highest R ^2^ and is easy to use in the clinic for ^18^ F-FDG PET/CT image quality assessment. The new FDG dose regimen based on the patient's body weight is recommended for new generations of highly sensitive scanners. For our highly sensitive BGO PET-CT scanner (Discovery IQ 5 ring), we proposed a new dose-time regimen based on body weight that can significantly reduce the injection dose and scan times while maintaining sufficient image quality for diagnosis.
